# High density lipoprotein cholesterol and proteome in SR-B1 KO mice: lost in precipitation

**DOI:** 10.1186/s12967-018-1683-4

**Published:** 2018-11-12

**Authors:** Susana Contreras-Duarte, Nicolás Santander, Ruth Birner-Gruenberger, Christian Wadsack, Attilio Rigotti, Dolores Busso

**Affiliations:** 1 Department of Nutrition, Diabetes and Metabolism, Pontificia Universidad Católica de Chile, Santiago, Chile; 2 Institute of Pathology and Center of Medical Research, Medical University of Graz, Graz, Austria; 3 Austrian Center of Industrial Biotechnology, Medical University of Graz, Graz, Austria; 4 Omics Center, Graz, Austria; 5 Department of Obstetrics and Gynecology, Medical University of Graz, Graz, Austria

**Keywords:** HDL, Lipoproteins, Proteomics, SR-B1 KO mice

## Abstract

Scavenger receptor class B type 1 (SR-B1) plays an essential role in high density lipoprotein (HDL) metabolism. SR-B1 deficient (SR-B1 KO) mice are prone to atherosclerosis and exhibit abnormally large, cholesterol-rich, dysfunctional HDL. In a recent issue of J Transl Med, Cao et al. described results of proteomics analyses of HDL isolated from wild-type (WT) and SR-B1 KO mice using precipitation of large lipoproteins with polyethylene glycol (PEG). They report abnormalities in SR-B1 KO HDL protein components that correlate with HDL function. In this commentary, we describe and discuss the differences in the results published by Cao et al. and those obtained in a recent study from our laboratory using shotgun proteomics of HDL of SR-B1 KO mice isolated by ultracentrifugation. We propose that different HDL purification procedures used may account for the discrepancies observed. We show that SR-B1 KO HDL purification using either PEG or dextran sulfate precipitation results in enrichment of small HDL subclasses, and may therefore underestimate alterations in lipoprotein composition or function. Compared to HDL obtained by ultracentrifugation, HDL isolated by PEG precipitation show a lower ApoE/ApoA-I proportion and reduced cholesterol content. HDL protein components described by Cao et al. or our laboratory are mostly inconsistent: only 33 HDL proteins were detected in both datasets, whereas a significant number of proteins were only identified by Cao et al. (n = 43) or Contreras-Duarte et al. (n = 26) datasets. The relative abundance of HDL-associated peptide and protein levels in WT vs SR-B1 HDL were also highly different in both datasets. This study indicates that caution must be taken when interpreting results from HDL isolated by chemical precipitation.

**Electronic supplementary material:**

The online version of this article (10.1186/s12967-018-1683-4) contains supplementary material, which is available to authorized users.

## Main text

In a recent issue of the *Journal of Translational Medicine*, Cao et al. [1] studied the proteome associated with high density lipoproteins (HDL) isolated from scavenger receptor class B type 1 deficient mice (SR-B1 KO). SR-B1 plays an essential role in HDL-mediated reverse cholesterol transport, so SR-B1 KO mice have abnormally large, cholesterol-rich dysfunctional HDL and are prone to atherosclerosis [2, 3].

In their work, Cao et al. show changes in the protein content of SR-B1 KO HDL that may be associated with alterations in lipoprotein functionality. Specifically, they describe lower levels of proteins involved in lipid metabolism and redox regulation as well as higher levels of proteins related to inflammatory processes and proteinase modulation compared to WT animals. Such changes in the HDL proteome were associated with differences in several biological activities commonly attributed to HDL particles: HDL from SR-B1 KO mice showed reduced cholesterol efflux capacity, reduced antioxidant activity, and they were less anti-inflammatory than HDL from wild-type (WT) mice. Finally, the authors suggest that some specific proteins associated with HDL particles may be used as diagnosis biomarkers, potentially useful for the identification of individuals with dysfunctional SR-B1-mediated HDL metabolism.

Current evidence shows that HDL purification methods may lead to significant modification in the composition and function of these lipoproteins [4]. In this commentary, we compared the protein composition of WT and SR-B1 KO mouse HDL obtained by two different isolation methods: (1) data from Cao et al. using HDL purified after precipitation of non HDL lipoproteins with polyethylene glycol (PEG), a neutral polymer that reduces the solubility of apolipoprotein B (ApoB)-containing lipoproteins, and (2) our own shotgun proteomics results of HDL particles obtained by ultracentrifugation. The comparative analysis shows important differences between protein compositions obtained in each of the above studies, and suggests that ApoB precipitation may lead to underestimation of some HDL protein components.

The precipitation procedure with PEG used by Cao et al. has been used extensively because it is a fast and easy method to isolate HDL from whole plasma or serum, thus it is employed routinely to measure HDL cholesterol in clinical settings [5]. A recent study comparing different methods for precipitation of large lipoproteins before HDL analysis reported that PEG precipitation induced a profound rightward shift into smaller particles in the elution profile of HDL after gel filtration. This modification of HDL particle size due to PEG use may be caused by the specific dissociation of certain proteins from HDL or to precipitation of larger HDL particles that are closer to the density and/or other physicochemical properties of LDL [6].

**Fig. 1:**
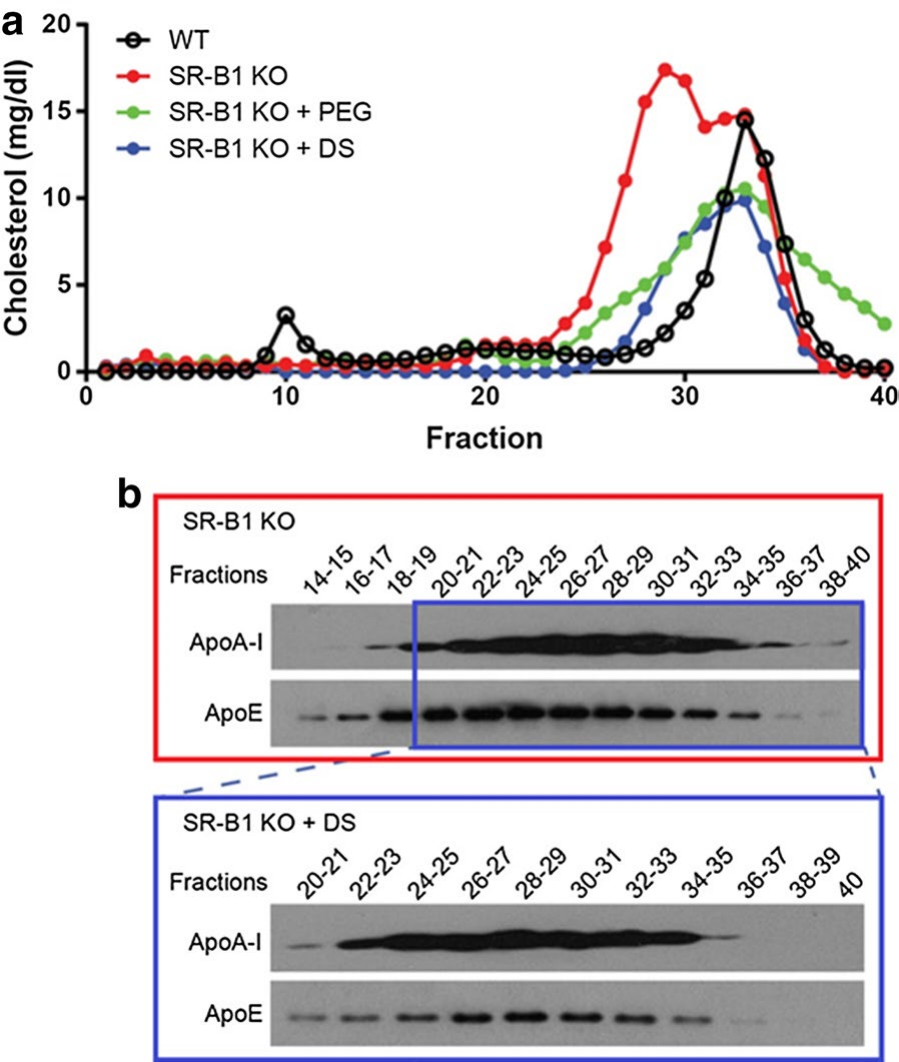
Alterations in HDL particles after isolation from plasma by chemical precipitation of non HDL lipoproteins. **a** Plasma from wild-type (WT) or SR-B1 KO animals and plasma pre-treated with polyethylene glycol (PEG) or dextran sulfate (DS) were fractionated by fast protein liquid chromatography (FPLC, Superose-6 column, GE Life Sciences, PA) and cholesterol was determined in each fraction. **b** Levels of ApoA-I and ApoE were determined by western blotting in the fractions obtained by FPLC separation (**a**)

SR-B1 KO mice have large, cholesterol-rich, dysfunctional HDL [2]. We recently evaluated the effectiveness of two purification strategies using precipitation (PEG versus dextran sulfate [DS]) to isolate HDL from WT and SR-B1 KO mice. We observed that in both methods a striking rightward shift was observed in the HDL size exclusion chromatography elution profile (Fig. 1a), as previously reported for HDL from healthy human subjects [4]. Thus, after using either precipitation method, the HDL present in the supernatant are enriched in smaller HDL particles and/or represent HDL particles modified in their hydrodynamic properties, potentially caused by changes in their molecular composition. Western blotting studies of fractions corresponding to normal HDL-sized particles eluted from the fast protein liquid chromatography column showed that, compared to total plasma, DS-purified HDL had a lower ApoE/ApoA-I ratio and eluted in fractions containing smaller lipoproteins (Fig. 1b). These results indicate that lipoprotein precipitation as a purification method to isolate HDL from SR-B1 KO plasma may underestimate alterations in HDL protein composition and in their functionality. Also, HDL cholesterol levels are underestimated after HDL purification by precipitation, as shown by the reduction in the areas under the curves of cholesterol chromatographic distribution in Fig. 1a. Our results show that these precipitation methods, used routinely in clinical labs to measure HDL cholesterol, may generate misleading values when applied to subjects with abnormally large HDL particles.

In order to assess differences in protein components between HDL from WT and SR-B1 KO mice, we isolated HDL by ultracentrifugation from serum of mice of both genotypes. Our results showed that 17 proteins were overrepresented and one protein was underrepresented in HDL from SR-B1 KO compared to HDL from WT mice (Additional file 1). The upregulated proteins were functionally related to lipid and lipoprotein metabolism (lipoprotein particle remodeling, reverse cholesterol transport, cholesterol homeostasis, lipoprotein metabolic process, positive regulation of lipid biosynthetic process), whereas some of them were also related to additional functions/processes such as cellular response to iron, hydrogen peroxide catabolism, blood coagulation, and regulation of endocytosis.

**Fig. 2:**
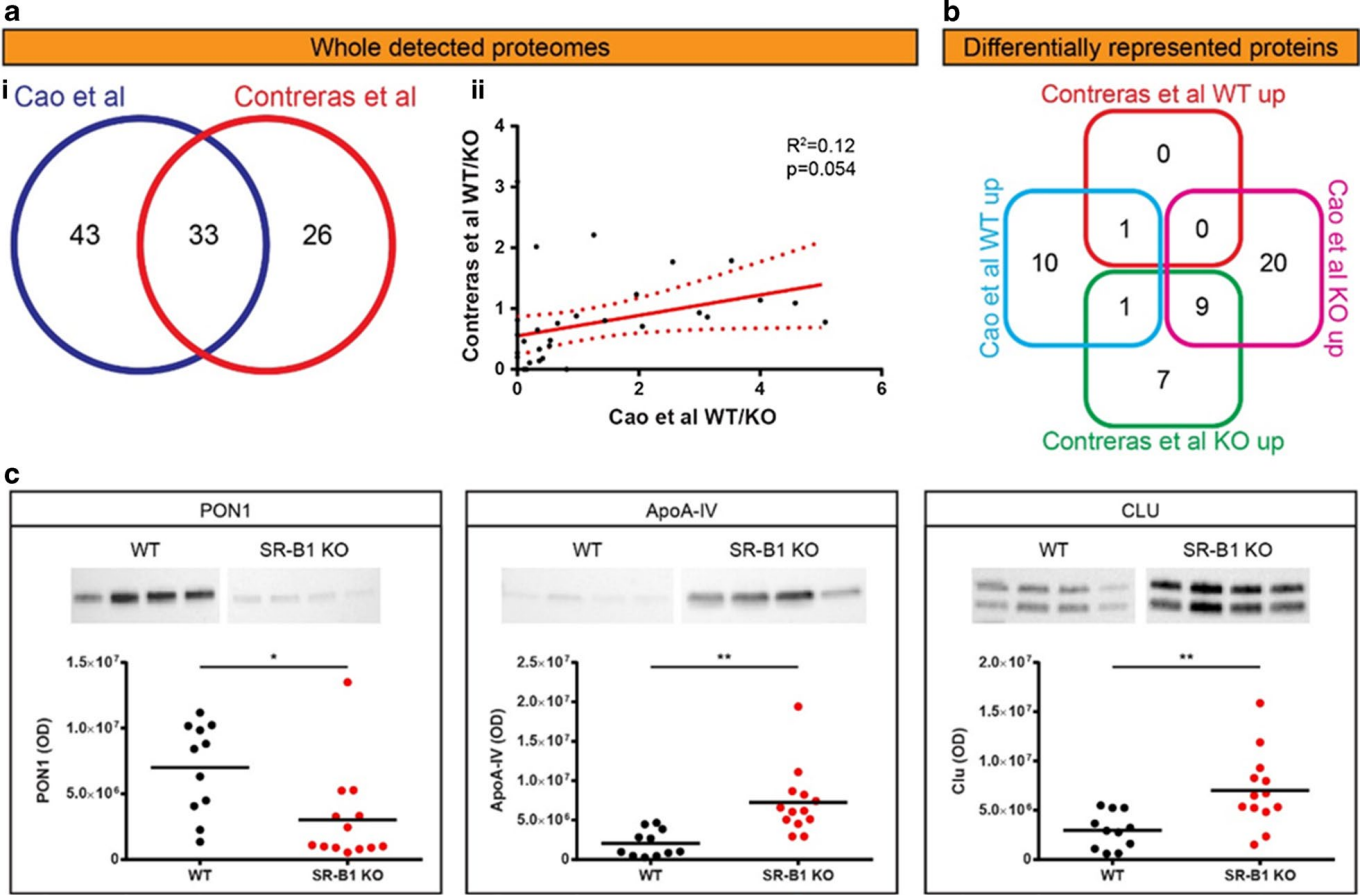
Comparative proteomic analyses of HDL obtained from WT and SR-B1 KO mice. **a.i** Qualitative changes in WT and KO HDL-associated proteins detected in the dataset by Cao et al. [1] and in our dataset. **a.ii** Correlation analysis of the levels of HDL-associated proteins detected in both datasets. **b** Analysis of HDL-associated proteins showing higher abundance (up) in WT or SR-B1 KO mice in both datasets. **c** Abundance of selected proteins found to be differentially associated with HDL in WT (n = 11) and SR-B1 KO (n = 13) mice by proteomics was further evaluated by Western blot and bands were quantitated by densitometry using Image J 1.45 Software. *p < 0.05, **p < 0.01

The comparison between our dataset and the one published by Cao et al. [1] (an editable version of their dataset in Additional file 2) revealed important qualitative and quantitative differences between proteins in SR-B1 KO vs. WT HDL. First, only 33 proteins were detected in both datasets, and a significant number of proteins were only detected by Cao et al. (n = 43) or by Contreras-Duarte et al. (n = 26) (Fig. 2a.i). Second, among proteins consistently detected in both datasets, the differences between WT and SR-B1 KO HDL only showed a weak correlation (Fig. 2a.ii). Third, proteins showing statistically significant differences between WT and SR-B1 KO HDL demonstrated little consistency in the two datasets: only ten proteins out of 48 proteins showed reproducible changes in both studies (Fig. 2b).

In order to validate the shotgun proteomics results, we analyzed by western blotting the levels of three proteins in HDL samples from WT and SR-B1 KO mice isolated by ultracentrifugation: [1] Apolipoprotein A-IV (ApoA-IV), overrepresented in HDL from SR-B1 KO mice in both datasets; [2] Paraoxonase 1 (PON1), only reduced in SR-B1 KO-derived HDL in the dataset by Cao et al. and [3] Clusterin (CLU, also known as ApoJ), only overrepresented in our SR-B1 KO dataset. The results of this study showed that PON1 levels were lower in HDL from SR-B1 KO than from WT mice (Fig. 2c) whereas ApoA-IV and CLU levels were higher in HDL from KO than WT mice (Fig. 2c). The use of plasma samples from different mice cohorts and/or the higher sensitivity of western blotting to detect differences between protein levels in WT and SR-B1 KO HDL may explain these differences.

Our preliminary results described in this commentary show that precipitation methods for HDL isolation might lead to alterations in the proportions of HDL subpopulations and/or HDL protein components, and suggest that this effect may be even larger during isolation of abnormal HDL particles with alterations in size and/or composition. In a clinical setting, HDL particle size in plasma, evaluated by nuclear magnetic resonance, has been associated directly with coronary artery disease risk [7] and inversely with insulin sensitivity [8]. Since large HDL may be lost along with ApoB-containing lipoproteins after chemical precipitation, using methods that avoid this isolation procedure may be required for initial characterization of HDL composition and functionality, particularly under pathological conditions or when evaluating new drugs that modulate HDL metabolism [9].

The isolation of HDL by precipitation may also lead to underestimation of HDL cholesterol due to precipitation of large, cholesterol-rich HDL particles. For example, in studies aimed at understanding the impact of SR-B1 biology on human HDL metabolism and coronary heart disease risk by identification of functional mutations in the *SCARB1* gene (coding for human SR-B1), patients with HDL cholesterol levels above the 95th percentile were selected for targeted sequencing [10]. However, since clinical laboratories usually inform HDL cholesterol levels after precipitating ApoB-containing lipoproteins, this strategy probably underestimates HDL cholesterol levels in subjects carrying *SCARB1* mutations, similar to what we showed after isolating HDL by precipitation of SR-B1 KO mouse plasma (Fig. 1a). A similar situation may occur when screening and/or evaluating patients with high HDL cholesterol levels due to CETP deficiency [11]. Thus, this routine methodology might preclude finding subjects with relevant mutations that lead to very large HDL, with excess cholesterol content, because abnormal lipoproteins present in these patients will be discarded by precipitation together with VLDL and LDL.

We propose that the HDL isolation method must be carefully considered during the characterization of HDL composition and functional properties, in particular in conditions were HDL are most prone to undergo a shift to large HDL particles. Disregarding this issue may have negative consequences not only in the identification of novel biomarkers of HDL functionality, but also in the design of studies and screening criteria involving patients with abnormal HDL structure or function or when evaluating new HDL-targeted therapies.

## Additional files

**Additional file 1.** Spectral counts obtained of HDL purified by ultracentrifugation from pools of plasma from 3 to 4 mice.

**Additional file 2.** Dataset generated by Cao et al. “Protein markers of dysfunctional HDL in scavenger receptor class B type I deficient mice”. J Transl Med 2018 16:155.

## Abbreviations

SR-B1 KO: scavenger receptor class B type 1 knock out mice
WT: wild type
HDL: high density lipoprotein
PEG: polyethylene glycol
DS: dextran sulfate
Apo: apolipoprotein
